# 1T Pixel Using Floating-Body MOSFET for CMOS Image Sensors

**DOI:** 10.3390/s90100131

**Published:** 2009-01-07

**Authors:** Guo-Neng Lu, Arnaud Tournier, François Roy, Benoît Deschamps

**Affiliations:** 1 Institut des Nanotechnologies de Lyon (INL), CNRS UMR5270, Université Claude Bernard Lyon1, 43 Bd du 11 Novembre 1918, 69622 Villeurbanne Cedex, France; 2 STMicroelectronics, Front-End Technology and Manufacturing (FTM), 850 rue Jean Monnet, 38926 Crolles Cedex, France; E-Mails: arnaud.tournier@st.com; françois.roy@st.com; benoit.deschamps@st.com

**Keywords:** 1T pixel, CMOS image sensors (CIS), floating-gate MOSFET, modeling, rectangular-gate pixel, ring-gate pixel

## Abstract

We present a single-transistor pixel for CMOS image sensors (CIS). It is a floating-body MOSFET structure, which is used as photo-sensing device and source-follower transistor, and can be controlled to store and evacuate charges. Our investigation into this 1T pixel structure includes modeling to obtain analytical description of conversion gain. Model validation has been done by comparing theoretical predictions and experimental results. On the other hand, the 1T pixel structure has been implemented in different configurations, including rectangular-gate and ring-gate designs, and variations of oxidation parameters for the fabrication process. The pixel characteristics are presented and discussed.

## 1. Introduction

In recent R&D efforts on CMOS image sensors (CIS), there have been many attempts to reduce pixel size for higher image resolution and/or higher density of integration. One obvious way to do this is to minimize the number of in-pixel transistors. This has led to architectural explorations instead of accepting 3 T and 4 T pixel structures as standard. Sharing pixel transistors has been proposed [1-3] and proved to be an effectively approach: it allows the number of transistors per pixel to be reduced to 2.5 T, 1.75 T, or 1.5 T. On the other hand, a more ambitious approach aims at ultimate achievement: single component for the pixel. There have been suggestions of specific transistor structures as single pixel component working on the charge-modulation principle [4-6]. However, to meet system integration requirements, the structure of the pixel transistor should be simple, compact, and integrable with minimum extra fabrication process steps.

This paper presents a 1T pixel using a floating-body MOSFET. Instead of employing a photodiode (PD), the transistor is also operated as photo-sensing device, with its floating body to collect and store charges during integration. For signal readout, the transistor is operated as source follower. Finally for reset operation, the stored charges can be evacuated by bias control. We describe this 1T pixel structure and its operating principle in the following section (Section 2). Our studies include device modeling and model validation (in Section 3), pixel implementation (in Section 4), as well as characterization. The evaluated performances are presented in Section 5.

## 2. Pixel Structure and Operating Principle

The proposed 1T pixel consists of an n-type MOSFET. Alternatively a p-type MOSFET may also be used. However, n-type transistor pixel operates with collection and storage of photo-generated holes rather than electrons, which is a better choice to reduce electrical crosstalk, because holes have much lower mobility than electrons.

The transistor structure differs from the conventional one mainly in that floating P-well with controlled doping profile is employed. It is also used as photo-sensing element of the pixel. At the same time, under different bias conditions, this same transistor can be operated to perform integration, readout and reset.

**Figure 1.**
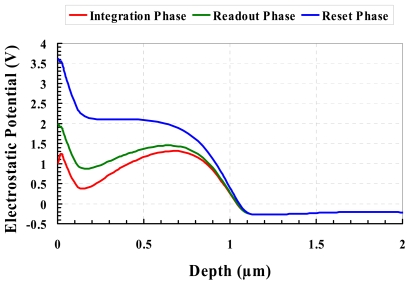
Potential profile of the transistor body.

In the integration phase, a low voltage level is applied to the gate of the transistor to turn it off. The floating body of the transistor exhibits a potential valley for collecting and storing holes (red curve in Figure 1). The pixel being under illumination, light penetrates through the gate and is absorbed in the transistor body. There is separation of photo-generated electron-hole pairs due to built-in electrical field in the body. The electrons are swept away mainly to the *V_dd_*-connected drain, while the holes are collected and accumulated in the potential valley. The stored holes raise the potential of the transistor body, which leads to a decrease of the transistor threshold voltage *V_tn_* [6].

In the readout phase, the transistor is switched on by applying a gate voltage higher than the maximum V_tn_ corresponding to the dark conditions. The potential valley becomes shallower with shrink of charge-storage region under the transistor gate (green curve in Figure 1). The transistor in this phase is operated as a source follower with fixed gate and drain voltages. The decrease of V_tn_ reflecting the amount of the stored charge is sensed as an increase of the transistor's effective gate-source voltage. Accordingly, the drain current and the output source voltage increase. The source voltage in this period can be readout by sampling.

For the reset of the pixel, a still higher voltage is applied to the transistor gate. At the same time, the source voltage is clamped to the drain voltage to minimize channel current. The potential valley disappears and the stored holes are pushed away to the substrate because the transistor body potential near the silicon surface is so substantially increased that the potential becomes monotonic decreasing (blue curve in Figure 1).

**Figure 2.**
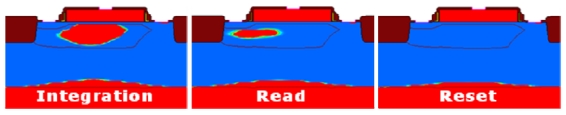
Simulations of the 1T pixel using ISE TCAD tools to verify its operation in different phases.

The operation of the 1T pixel can be verified by simulations using ISE TCAD tools, as is shown in Figure 2. The biasing conditions of the transistor are indicated in Table 1.

**Figure 3.**
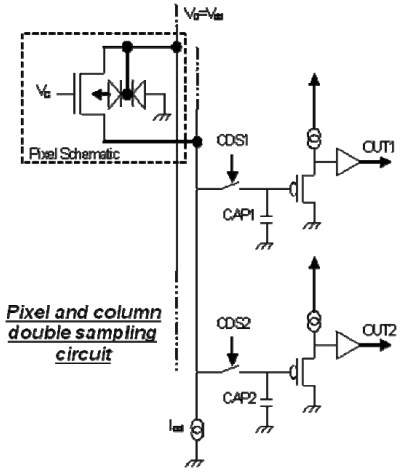
Readout by simple non-correlated double sampling.

One feature of this 1T pixel is its reset mode with depletion of the storage zone does not generate kTC noise. Thus there is no need to employ CDS (correlated double sampling). Instead, rapid simple non-correlated double sampling is suggested (see Figure 3) to suppress *V_tn_* dispersion and thus to reduce FPN (fixed pattern noise). It is more efficient in reducing low-frequency noise.

One should notice that in readout mode, some stored holes are displaced under the source due to shrink of charge storage region under the gate and source potential lowering. This shift of the charge storage region to the source and toward the pixel edge should be stopped to avoid electrical crosstalk. One solution to the problem is to employ STI (shallow trench isolation), as shown in Figure 2. It was adopted in our first design configuration.

## 3. Modeling and Model Validation

### 3.1. Model Description

For optimizing performances of the 1T pixel, we have built a model allowing determination of linear conversion characteristics such as conversion gain (CG). The modeling approach is described as follows.

**Figure 4.**
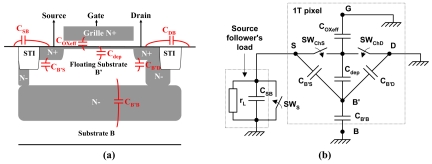
**(a)** 1T pixel structure with indication of capacitances. **(b)** Its ac equivalent circuit.

The first step is to consider the capacitances of the pixel structure and to establish an ac equivalent circuit for estimating the total capacitance of the transistor's floating body. It is this capacitance that plays the role of charge storage and determines the relationship between stored holes and the potential of the floating body. Figure 4a shows the pixel structure with inherent capacitances. The pixel transistor has, beside its drain, gate, source and bulk having external connections, two internal nodes: its channel C and its floating body B' where holes can be stored in integration and readout modes. Figure 4b presents the corresponding ac equivalent circuit for both integration and readout operations. It does not include drain-ground and gate-ground capacitances because they are short-circuited by bias voltages. The switching positions of *S_WChS_*, *S_WChD_* and *SW_S_* of the equivalent circuit depend on the operating modes. In integration mode, as the transistor is off, both *SW_ChS_* and *SW_ChD_* are thus open. In readout mode, *SW_ChS_* and *SW_ChD_* are closed because the transistor is on (in saturation). It may look oversimplifying to assume the same potential for the source, the channel and the drain, but the situation is that the channel resistance of the on-state transistor is negligible (∼ 10^6^ Ω) compared to the impedances of the in-pixel capacitances (∼ 10^10^ Ω) at operating frequencies, and that *C_OXeff_* is effectively “short-circuited” by at least half of the channel resistance in parallel with it. Another way to see it is that the channel resistance imposes the C node to much lower impedance, like a “shield” to prevent effect of *C_OXeff_*. For *SW_S_*, it is open only in readout mode.

From the equivalent circuit of Figure 4b, the total capacitance of the floating body *B'*, denoted *C_B'_*, can be written as:

(1) $$C_{B\prime }=\{\begin{array}{c} \begin{array}{cc} \frac{C_{\text{OXeff}}C_{\text{dep}}}{C_{\text{OXeff}}+C_{\text{dep}}}+C_{B\prime S}+C_{B\prime D}+C_{B\prime B} & \left(\text{Integration}\right) \end{array}\\ \begin{array}{cc} C_{\text{dep}}+C_{B\prime S}+C_{B\prime D}+C_{B\prime B} & \left(\text{readout}\right) \end{array} \end{array}$$

It should be noted that in reset mode, when the stored charges are (normally) completely evacuated, *C_B_*_'_can be considered to be short-circuited. What is more important is readout-mode *C_B_*_'_, because it is related to the readout-determined conversion gain (CG) of the pixel, which will be expressed in (7).

The next step is to determine the pixel's linear conversion characteristics. The following describes how the conversion gain is determined. It is defined as:

(2) $$CG=\frac{\Delta V_{\text{pix}}}{\Delta N_{\text{ch}}}=\frac{1}{q}\frac{\Delta V_{\text{pix}}}{\Delta Q_{\text{ph}}}$$

where *V_pix_* is the pixel output voltage, N_ch_ the number of the collected photo-generated charges being stored in the pixel's integration capacitance, and *Q_ph_* the quantity of the stored charges. For this 1T pixel, the integration capacitance is no other than *C_B_*_'_. Thus the conversion gain can be expressed as:

(3) $$CG=\frac{1}{qC_{B\prime }}\frac{\Delta V_{\text{pix}}}{\Delta V_{B\prime }}$$

On the other hand, a variation of floating body potential (due to stored charges) will induce a shift of the transistor's threshold voltage *V_tn_*, which can be seen from the following relationship [7]:

(4) $$V_{\text{tn}}=\Phi_{\text{MS}}-2\Phi_{F}-\frac{\sqrt{2{ɛ}_{0}{ɛ}_{\text{Si}}qN_{B\prime }\left(2\Phi_{F}+V_{B\prime }-V_{S}\right)}}{C_{\text{OXeff}}/A_{G}}-\frac{Q_{\text{ox}}+Q_{\text{shal}}}{C_{\text{OXeff}}/A_{G}}$$

where *Φ_MS_* is the work-function difference, *2Φ_F_* is the surface inversion potential (which is 2 times the difference between the Fermi level of the substrate and intrinsic silicon), *N_B_* the doping concentration of the transistor's (floating) body, *V_B_*_'_the floating-body potential, *V_S_* the transistor's source potential, *C_OXeff_* the effective gate-oxide capacitance, *A_G_* the effective gate area, *Q_ox_* the oxide charge density and *Q_shal_* the shallow-implant charge density.

The derivative of *V_tn_* with respect to *V_B_*_'_ gives:

(5a) $$\Delta V_{\text{tn}}=-\frac{A_{G}}{2C_{\text{ox}}}\sqrt{\frac{2{ɛ}_{0}{ɛ}_{\text{Si}}qN_{B\prime }}{2\Phi_{F}+V_{B\prime }-V_{S}}}\Delta V_{B\prime }=-\frac{C_{\text{dep}}}{C_{\text{ox}}}\Delta V_{B\prime }$$

with:

(5b) $$C_{\text{dep}}=\frac{A_{G}{ɛ}_{0}{ɛ}_{\text{Si}}}{X_{\text{dep}}}$$

and:

(5c) $$X_{\text{dep}}=\frac{\sqrt{2{ɛ}_{0}{ɛ}_{\text{Si}}\left(2\Phi_{F}+V_{B\prime }-V_{S}\right)}}{qN_{B\prime }}$$

where *X_dep_* is the depletion-layer thickness between the transistor's channel and its floating body. It can be seen from (5) that increasing the floating body potential causes a lowering of the threshold voltage.

As the transistor in readout mode is biased with a constant gate voltage, and the drain current is function of (*V_G_* – *V_S_* – *V_tn_*), a decrease of *V_tn_* amounts to an equivalent increase in *V_G_*, i.e. – Δ*V_tn_* = Δ*V_G_*. Then, according to the source follower operation, we have:

(6) $$\Delta V_{\text{pix}}=\Delta V_{G}A_{v}=-\Delta V_{\text{tn}}A_{v}$$

where *A_v_* (∼ 0.9) is the source follower's voltage gain.

From (3), (5) and (6), we can rewrite the conversion gain as:

(7) $$CG=\frac{qA_{v}C_{\text{dep}}}{C_{\text{OXeff}}C_{B\prime }}$$

Finally this model may integrate models of involved parameters. For example, *C_dep_* in the above expression is related to the silicon surface potential, which in turn depends on properties of gate oxide and Si/SiO_2_ interface. We have modeled *C_dep_* based on analytical descriptions in [8, 9]. This has enabled us to analyze effects of process parameters.

It should be mentioned that capacitances in the silicon are voltage-dependent, especially when they result from lightly-doped regions, such as *C_SB_*_'_, *C_B'D_* and *C_B'B_*. It implies that the total capacitance *C_B_*_'_may vary with the floating body potential. Consequently, the conversion linearity may degrade. However, as will be seen in the following subsection, *C_SB'_, C_B'D_* and *C_B'B_* are much smaller than *C_dep_*. This means that *C_B_*_'_ ≈ *C_dep_*. We obtain thus a simplified expression of CG:

(8) $$CG\approx \frac{qA_{v}}{C_{\text{OXeff}}}$$

The above expression does not include capacitances in the silicon. Therefore, there are no significant effects of stored charges and induced potential variations on CG. The estimated linearity error of conversion is about 2%.

The expression (8) allows rough estimation of CG. It predicts (especially for process optimization) that CG may be improved by increasing the gate-oxide thickness *t_ox_*.

### 3.2. Extraction of Parameters

**Figure 5.**
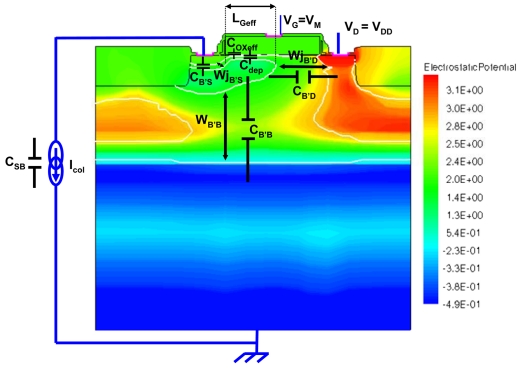
Simulated 1T pixel structure in readout phase with indication of parameters.

Calculating CG needs prior determination of the involved parameters in its expression. Via simulations using ISE TCAD tools, we can extract geometrical parameters to evaluate the pixel structure's inherent capacitances. Figure 5 shows one simulation example of the pixel structure operated in readout phase. Table 2 gives expressions of parameters and extracted values for a 2.2 μm-pitch 1 T pixel.

### 3.3. Model Validation

**Figure 6.**
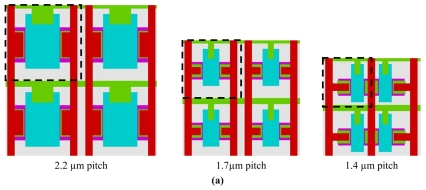

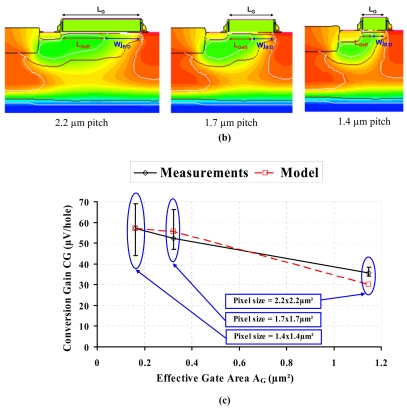
(a) Layout of three pixels in different sizes: 2.2 μm pitch, 1.7 μm pitch and 1.4 μm pitch. (b) Simulated structures of the three pixels. (c) Conversion gain against effective gate area.

We have performed model validation by comparing model predictions with measured results in parametrical analysis. Figure 6a presents the layout of 3 pixel sizes: 2.2 μm pitch, 1.7 μm pitch and 1.4μm pitch. Figure 6b shows the 3 simulated pixels (allowing extraction of geometrical parameters). The evaluated effective gate area *A_G_* (= *L_Geff_* × *W_Geff_*) for these pixel sizes is 1.15 μm^2^, 0.322 μm^2^ and 0.161 μm^2^ respectively. Figure 6c compares calculated and measured results on CG as a function of A_G_. Both aspects of results show an increase of CG when scaling down the pixel. However, this increase is much slower than what predicts the scaling law, according to which CG would be inversely proportional to the pixel area. This comparison of results shows that the prediction from the model is fairly accurate. The fact of some involved capacitances that scale slowly may account for this CG evolution.

**Figure 7.**
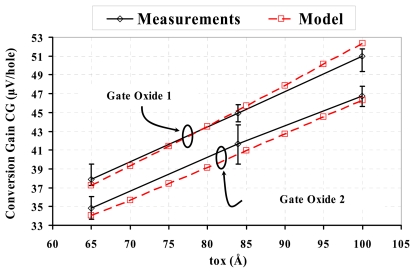
Conversion gain versus gate oxide thickness for two configurations of oxidation process: oxidation with nitridation (gate oxide 1) and oxidation only (gate oxide 2).

Figure 7 plots calculated and measured results on CG versus gate-oxide thickness: *t_ox_* = 65 Å, 84 Å and 100 Å respectively. Two configurations of oxidation process are compared: one is oxidation plus nitridation (gate oxide 1), and the other is oxidation only (gate oxide 2). It has been reported [10, 11] that gate-oxide nitridation leads to a much higher density of Si/SiO_2_ interface traps. The charged interface states induces a shift of surface potential, which causes *C_dep_* to change. Accordingly CG may alter. The observed agreement between model prediction and experimental measurements confirms the validity of the model.

## 4. Implementation Configurations

Conventional design of the 1T pixel is the rectangular-gate configuration, as shown in Figure 6a. Implementing the 1T pixel in rectangular-gate configuration has minimized transistor size. However as already mentioned, to avoid electrical crosstalk we should employ STI. This solution unavoidably increases pixel size. Moreover, it also increase significantly the pixel dark current, because of increased silicon surface depletion areas, where dark-current generation is a major contribution [12].

**Figure 8.**
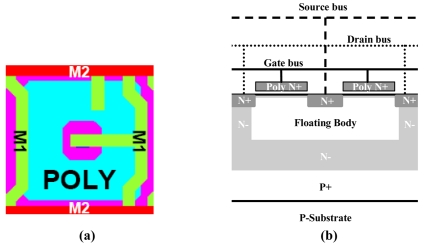
Ring-gate design of the 1 T pixel structure in a 0.13 μm CMOS process & 90 nm copper-based process. (a) 1.4 μm-pitch pixel layout. (b) Cross-section view of the implemented pixel structure.

Another way of designing 1T pixel is a ring-gate configuration, with source at the center and peripheral drain (shown in Figure 8a). This time the readout-induced shift of the charge-storage region goes to the pixel center, which does not raise crosstalk problems. Thus there is no need to employ STI. The floating body of the transistor is shielded by a deep buried N-type layer and N peripheral region below the drain (see Figure 8b). The surrounding drain and the non-depleted N-peripheral region underneath in the peripheral pixel area also play the role of pixel isolation. This STI suppression allows not only smaller pixel size and/or fill-factor improvement, but also substantial reduction of surface dark current component.

At the fabrication process level, we have implemented several configurations on test chips with variations of parameters, with the aim of both model validation (subsection 3.3.) and optimization of pixel characteristics. This includes:

- increasing gate-oxide thickness to enhance CG;
- gate oxidation with and without nitridation (gate oxide 1 and gate oxide 2) to choose one with better noise performance.

As gate-oxide nitridation may induce much more interface traps, a higher level of low-frequency noise in the MOS transistor is predicted. The interface-trap-related noise includes RTS (Random Telegraph Signal) noise and 1/f noise due to both carrier-trap (ΔN) and charge-scattering (Δμ) effects [13-15]. The low-frequency noise is becoming an important issue for CMOS image sensors (CIS) as the size of transistor components continues to shrink [16]. It may have dominant contribution to pixel read noise, and thus should be taken into account in the choice of oxidation parameters.

It should be mentioned that stored holes in the pixel transistor's floating body will not communicate with interface traps, and that the main noise effect of these traps is resulting fluctuations of the transistor drain current when it is in readout mode.

## 5. Pixel Characteristics

Measurements of test chips have been made to estimate impacts of oxidation process parameters on CG (shown in Figure 7) and on temporal noise. Temporal noise of the pixel structure has been evaluated by measuring its output fluctuations in dark conditions with short integration duration, so as to neglect dark-current-contributed shot noise. The measured results shown in Table 3 confirm noise lowering with the increase of *t_ox_*, because increasing *t_ox_* will enhance CG, and thus lead to lower equivalent noise. On the other hand, the observed difference of noise levels between oxidation with and without nitridation indicates that interface-trap-induced noise is a main temporal noise source.

**Figure 9.**
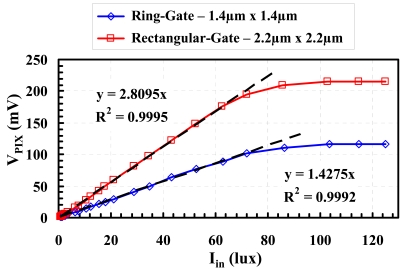
Photo-conversion transfer characteristic curves of two design configurations: 2.2 μm-pitch rectangular-gate pixel and 1.4 μm-pitch ring-gate pixel respectively.

Figure 9 shows two photo-conversion transfer characteristic curves, corresponding to a 2.2 μm-pitch rectangular-gate pixel and a 1.4 μm-pitch ring-gate pixel respectively. From the photoelectric conversion characteristic curve *V_pix_*(*I_in_*), several performance aspects can be evaluated: the first portion of the curve before saturation determines the sensitivity and the linearity, while the saturation level indicates the full-well capacity (FWC).

The sensitivity corresponds to the slope of the curve divided by the integration time *t_int_*:

(9) $$S=\frac{1}{t_{\text{int}}}\frac{\Delta V_{\text{pix}}}{\Delta I_{\text{in}}}$$

It can be shown to be geometry-dependent, and proportional to the pixel's sensing surface area as well as its conversion gain. Table 4 presents measured sensitivity of different design configurations. The obtained results show that:

- pixel shrink will lead to rapid degradation of sensitivity;
- for a given pixel size the ring-gate configuration has better sensitivity than the rectangular-gate one.

Table 5 compares characteristics of two fabricated CIS test chips, one integrating an array of 2.2 μm-pitch rectangular-gate pixels [17], and the other an array of 1.4μm-pitch ring-gate pixels [18]. The pixel fabrication process requires only three extra masks for specific implants and is fully compatible with the CMOS digital process.

The 1.4 μm-pitch ring-gate pixel has improved characteristics compared to its 2.2 μm-pitch rectangular-gate counterpart:

- smaller size for a comparable fill factor, mainly because STI is not employed;
- larger CG, because of smaller size;
- much lower dark current thanks to STI suppression, smooth-shape layout and smaller size;
- lower temporal noise, partly because of CG improvement;
- much lower Dark FPN (Fixed Pattern Noise, which may in large part be due to dark current), thanks to dark current reduction;
- larger dynamic range, because improved signal-to-noise ratio outweighs FWC degradation.

The 1.4μm-pitch pixel has also degraded performances:

- lower FWC, because of smaller size and ring shape of the charge-storage region [19];
- poorer sensitivity, due to size reduction.

**Figure 10.**
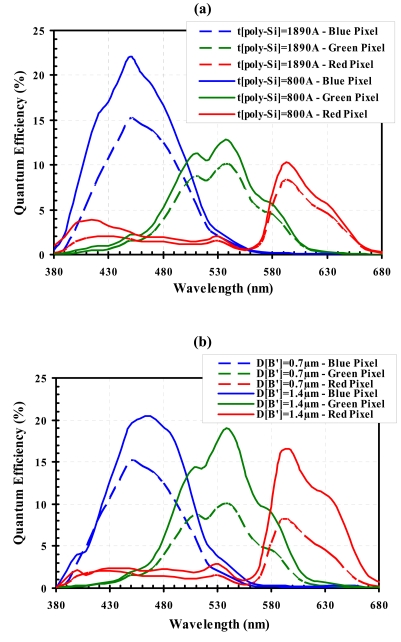
Simulated results on the pixel quantum efficiency (with color filters) for: (a) a poly-gate *t_poly-si_* = 800 Å (solid curves) compared to *t_poly-si_* = 1890 Å (dash curves); (b) charge-collecting region centered at depth *D_B'_*= 1.4 μm (solid curves) instead of *D_B_*_'_= 0.7 μm (dash curves).

The above comparison between the two design configurations shows that some performance aspects can substantially be enhanced by the use of appropriate design techniques. It is, however, a challenging task to preserve and/or improve FWC and sensitivity when reducing pixel size. Methods of improvements include optimization of process and bias parameters. Figure 10 presents two examples of quantum-efficiency (QE) improvement: employing thinner Poly-Si gate to reduce short-wavelength absorption loss (Figure 10a), and enlarging charge-colleting region by implant optimization (Figure 10b).

FWC of the 1T pixel may be improved by lowering *V_tn_* of the transistor via shallow channel implant, so as to widen the gate-bias-voltage difference between reset and readout modes (i.e. $V_{G}^{\text{Rst}}-V_{G}^{\text{Rd}}$). As can be seen from Figure 1, $V_{G}^{\text{Rd}}$ should be low enough for obtaining a potential valley with a certain depth, and $V_{G}^{\text{Rst}}$ should be high enough to sweep away completely stored holes. Decreasing *V_tn_* leads to the decrease of $V_{G}^{\text{Rd}}$, which means an increase of the term ( $V_{G}^{\text{Rst}}-V_{G}^{\text{Rd}}$). Another way of increasing the term ( $V_{G}^{\text{Rst}}-V_{G}^{\text{Rd}}$) is to consider possible higher supply voltages (for the thick-gate-oxide pixel). It should be noted that rectangular-gate configuration cannot benefit from this supply-voltage relaxation because of early appearance of band-to-band tunneling effect [17]. Ring-gate configuration, on the other hand, seems to withstand a higher supply voltage without sharp increase of dark current.

Via implant control, the potential profile of the pixel transistor can be optimized to increase the depth of the potential valley in readout mode, but this will also increase difficulties to ensure complete evacuation of stored holes in reset phase. It should be mentioned that *V_G_* is not the only controlling voltage: the perimeter of the charge-storage region depends on the transistor bias voltages *V_S_, V_G_* and *V_D_*. Especially *V_S_* has a more efficient control than *V_G_*, with:

(10) $$\overset{\partial W_{jB\prime S}}{\;}/\underset{\partial V_{S}^{\text{Rst}}}{\;}>>\overset{\partial X_{\text{dep}}}{\;}/\underset{\partial V_{G}^{\text{Rst}}}{\;}$$

Thus, combining optimization of bias and implant parameters may be an effective approach for FWC improvement.

## 6. Conclusions

We have proposed a floating-body MOSFET as a single pixel component. It can be operated as photo-sensing device and source-follower transistor, with charge storage and charge evacuation via bias control. Our investigation into this 1T pixel structure includes modeling and model validation, implementation and characterization.

The pixel structure has been modeled by establishing an equivalent circuit, which allows analytical description of the pixel's linear conversion characteristics. The relationship of the conversion gain with key parameters has thus been determined. The involved parameters have also been modeled and integrated in the device model to allow parametrical analysis. Model validation has been done by comparing theoretical predictions and experimental results.

The proposed pixel structure has been designed in rectangular-gate and ring-gate configurations. Due to stored charges moving toward the transistor's source, the former requires the use of STI to avoid electrical crosstalk, while the latter with the transistor's source at the pixel center suppresses this need. The implemented configurations on test chips include variations of oxidation process parameters for model validation and performance optimization.

The obtained results confirm that reducing pixel size improves conversion gain, but degrades full well capacity. Ring-gate pixel design has much lower dark current than the rectangular-gate counterpart, mainly thanks to STI suppression and smooth-shape layout. Moreover, the ring-gate pixel has lower noise and much lower dark FPN. Dark FPN may largely be contributed by dark current. The dynamic range for the ring-gate pixel is larger, meaning that signal-to-noise ratio outweighs FWC degradation. However, the sensitivity, like FWC, is also degraded in the same proportion. Possible improvements of performances include optimization of process and bias parameters.

## Figures and Tables

**Table 1.** Biasing conditions of the pixel transistor.

	**V_D_**	**V_G_**	**V_S_**
**Integration Readout**	3.3 V	∼ 0 V	3.3 V
	3.3 V	∼ 2 V	Connection to load
**Reset**	3.3 V	3.3 V	3.3 V

**Table 2.** Expressions of parameters and evaluated values for a 2.2 μm-pitch 1 T pixel.

**Parameter**	**Expression**	**Value**
**A_G_**	$A_{G}=L_{\text{Geff}}\times W_{\text{Geff}}$	0.62 μm^2^
**C_OXeff_**	$C_{\text{dep}}=\frac{A_{G}{ɛ}_{0}{ɛ}_{\text{ox}}}{t_{\text{ox}}}$	3.28 fF
**C_dep_**	$C_{\text{dep}}=\frac{A_{G}{ɛ}_{0}{ɛ}_{\text{Si}}}{X_{\text{dep}}}$	1.82 fF
**C_B'D_**	$C_{B\prime D}=\frac{Aj_{B\prime D}\cdot {ɛ}_{0}\cdot {ɛ}_{\text{si}}}{Wj_{B\prime D}}$	0.019 fF
**C_B'S_**	$C_{B\prime S}=\frac{Aj_{B\prime S}\cdot {ɛ}_{0}\cdot {ɛ}_{\text{si}}}{Wj_{B\prime S}}$	0.60 fF
**C_B'B_**	$C_{B\prime B}=\frac{A_{G_{\text{eff}}}\cdot {ɛ}_{0}\cdot {ɛ}_{\text{si}}}{W_{B\prime B}}$	0.081 fF
**C_B'_**	$C_{B\prime }=C_{\text{dep}}+C_{B\prime S}+C_{B\prime D}+C_{B\prime B}$	2.52 fF

This example shows that the 1T pixel structure has *C_oxeff_* ≫ *C_dep_* ≫ *C_SB'_, C_B'D_, C_B'B_*.

**Table 3.** Temporal noise (in equivalent holes) corresponding to different oxidation process parameters.

**t_ox_**	**65 Å**	**84 Å**	**100 Å**
**Ox + Ni (gate oxide 1)**	5.1 h^+^	4.9 h^+^	4.8 h^+^
**Ox only (gate oxide 2)**	4.5 h^+^	4.1 h^+^	4.0 h^+^

**Table 4.** Different design configurations and their sensitivity evaluated by measuring test chips.

	**Pixel size (μm^2^)**	**Fill factor**	**Sensitivity (h^+^/lux.s)**

**Rectangular-gate**	2.2 × 2.2 = 4.84	46%	1840
**Rectangular-gate**	1.7 × 1.7 = 2.89	40%	550
**Rectangular-gate**	1.4 × 1.4 = 1.96	34%	290
**Ring-gate**	1.4 × 1.4 = 1.96	50%	590

**Table 5.** Comparison of measured characteristics between the 2.2 μm-pitch rectangular-gate pixel and the 1.4 μm-pitch ring-gate pixel.

**Parameter**	**2.2μm-pitch rectangular-gate**	**1.4μm-pitch ring-gate**	**Testing conditions**

**Process**	0.13 μm 1 P 4 M CMOS	0.13 μm FE + 90 nm BE 1P 3M CMOS	
**Test chip size**	3.2 mm × 3.2 mm	3.0 mm × 3.2 mm	
**Pixel size**	2.2 μm × 2.2 μm	1.4 μm × 1.4 μm	
**Number of Pixels**	CIF (352 × 288)	VGA (672 × 512)	
**Fill factor**	46 %	50 %	Without microlens
**Supply voltage**	1.2 V / 3.3V	1.2 V / 3.3 V	
**Conversion gain**	35 μV/h^+^	58 μV/h^+^	
**Full well capacity**	6200 h^+^	2000 h^+^	
**Dark current**	500 h^+^/s	39.7 h^+^/s	Mean value @ RT
**Pixel temporal Noise**	6 h^+^	2.4 h^+^	In darkness
**Pixel Dark FPN**	39.5 h^+^	4.3 h^+^	Without additional correction circuit
**Noise floor**	40 h^+^	4.9 h^+^	Temporal noise, FPN & DSNU in darkness
**Dynamic range**	44 dB	52 dB	Usable Well over Noise floor
**Sensitivity**	1840 h^+^/lux.s	590 h^+^/lux.s	B/W sensitivity without microlens Halogen 3200 K IR cut off 650 nm
